# New C^4^D Sensor with a Simulated Inductor

**DOI:** 10.3390/s16020165

**Published:** 2016-01-27

**Authors:** Yingchao Lyu, Haifeng Ji, Shijie Yang, Zhiyao Huang, Baoliang Wang, Haiqing Li

**Affiliations:** State Key Laboratory of Industrial Control Technology, College of Control Science and Engineering, Zhejiang University, Hangzhou 310027, China; lychao1990@zju.edu.cn (Y.L.); jaky@cypress.com (S.Y.) zyhuang@iipc.zju.edu.cn (Z.H.); blwang@iipc.zju.edu.cn (B.W.); hqli@iipc.zju.edu.cn (H.L.)

**Keywords:** capacitively coupled contactless conductivity detection (C^4^D), contactless conductivity detection (CCD), series resonance, simulated inductor, conductivity measurement

## Abstract

A new capacitively coupled contactless conductivity detection (C^4^D) sensor with an improved simulated inductor is developed in this work. The improved simulated inductor is designed on the basis of the Riordan-type floating simulated inductor. With the improved simulated inductor, the negative influence of the coupling capacitances is overcome and the conductivity measurement is implemented by the series resonance principle. The conductivity measurement experiments are carried out in three pipes with different inner diameters of 3.0 mm, 4.6 mm and 6.4 mm, respectively. The experimental results show that the designs of the new C^4^D sensor and the improved simulated inductor are successful. The maximum relative error of the conductivity measurement is less than 5%. Compared with the C^4^D sensors using practical inductors, the measurement accuracy of the new C^4^D sensor is comparable. The research results also indicate that the adjustability of a simulated inductor can reduce the requirement for the AC source and guarantee the interchangeableness. Meanwhile, it is recommended that making the potential of one terminal of a simulated inductor stable is beneficial to the running stability. Furthermore, this work indirectly verifies the possibility and feasibility of the miniaturization of the C^4^D sensor by using the simulated inductor technique and lays a good foundation for future research work.

## 1. Introduction

Electrical conductivity is a key parameter of electrolyte solution. The on-line measurement of conductivity is of great importance to academic research and industrial applications [1,2,3,4]. Up to date, the conventional conductivity measurement techniques have mainly been based on contact conductivity detection. The direct contact between the electrodes and the solution may cause the polarization effect and the electrochemical reaction [5,6,7,8,9,10,11,12,13]. Meanwhile, the electrode contamination may bring about unpredictable measurement error. These drawbacks limit the applications of the contact conductivity detection technique.

**Figure 1:**
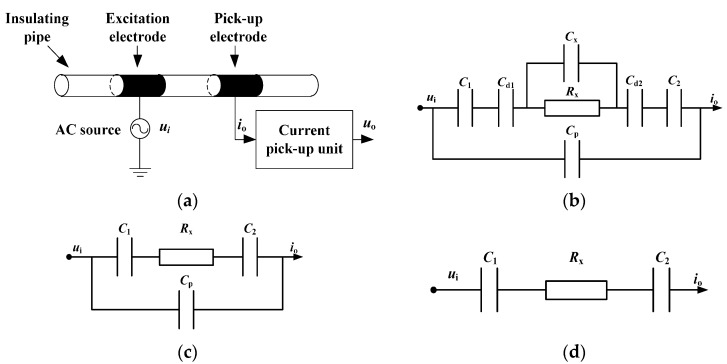
Principle of a typical C^4^D sensor: (**a**) Construction; (**b**) Equivalent circuit; (**c**) Simplified equivalent circuit; (**d**) Further simplified equivalent circuit.

The capacitively coupled contactless conductivity detection (C^4^D) technique is a capillary electrophoresis (CE) alternative conductivity detection method [5,6,7,8,9,10,11,12,13,14,15,16,17,18,19,20,21,22,23,24,25,26,27,28,29,30,31,32,33,34,35]. Its measurement principle can be briefly illustrated by Figure 1. As shown in Figure 1a, the construction of a typical C^4^D sensor includes two cylindrical metal electrodes fixed around the outside of an insulating pipe, an AC source and a current pick-up unit. Figure 1b shows the equivalent circuit of the typical C^4^D sensor. $C_{1}$ and $C_{2}$ are the coupling capacitances between the electrodes and the solution through the pipe wall. $C_{d1}$ and $C_{d2}$ are the electrical double-layer capacitances. $C_{p}$ is the stray capacitance. $C_{x}$ is the solution capacitance and $R_{x}$ is the resistor of the solution between the two electrodes. Research works have indicated that [3,7,8,9,10,11,12,13,14,15,16,17,18,19,20,21,22,23,24,25,26,27,28,31,32,33,34,35]: (1) The electrical double-layer capacitances $C_{d1}$ and $C_{d2}$ are in series with the coupling capacitances $C_{1}$ and $C_{2}$. The values of $C_{d1}$ and $C_{d2}$ are much larger than that of $C_{1}$ and $C_{2}$. Because the capacitance resulting from a series combination of large and small capacitances is determined essentially by the small capacitance, $C_{d1}$ and $C_{d2}$ can be ignored. (2) The solution capacitance $C_{x}$ is in parallel with the solution resisitor $R_{x}$. $C_{x}$ is of a very small order of magnitude (*i.e.*, the impedance of $C_{x}$ is much larger than that of $R_{x}$ in an AC path). Because the impedance resulting from a parallel combination is determined essentially by the small one, the influence of $C_{x}$ can be ignored. Thus, the influences of the electrical double-layer capacitances ($C_{d1}$ and $C_{d2}$) and the solution capacitance $C_{x}$ on the conductivity measurement are not significant and can be neglected. Additionally, the equivalent circuit Figure 1b can be simplified to Figure 1c which is the commonly used equivalent circuit in the C^4^D research field [7,8,9,10,11,12,17,18,20,21,22,23,24,25,26,27,31,32,33,34,35]. Meanwhile, research works have also verified that the negative influence of the stray capacitance $C_{p}$ can be overcome by introducing a grounded plane/shield [7,8,9,10,11,20,21,22,23,24,25,26,27,28]. Thus, the equivalent circuit Figure 1c can be further simplified to Figure 1d. In this work, Figure 1d is used for the following electrical circuit analysis and discussion because a grounded shield is introduced into our new C^4^D senor. When the AC source $u_{i}$ is applied, the current $i_{o}$ (which reflects the information of the solution resistor $R_{x}$) flows through the AC path to the current pick-up unit, and then the conductivity measurement is implemented.

Obviously, C^4^D is a contactless detection method. The polarization effect and the electrochemical reaction, which exist in the contact conductivity detection, can be avoided. So, the C^4^D technique has received considerable attention of scientists and engineers since it appeared [5,6,7,8,9,10,11,12,13,14,15,16,17,18,19,20,21,22,23,24,25,26,27,28,29,30,31,32,33,34,35]. However, to date, C^4^D has mainly been studied and applied in the research field of analytical chemistry for ion concentration/conductivity detection in a capillary [5,6,7,8,9,10,11,12,13,14,15,16,17,18,19,20,21,22,23,24,25,26,27,28,29,30,31,32,33,34,35]. It is still a developing technique, and its resolution and detection range should be improved. As shown in Figure 1d, in the view of electrical impedance measurement, only the resistor $R_{x}$ is the useful signal. Although the existence of the coupling capacitances $C_{1}$ and $C_{2}$ makes the contactless conductivity detection possible, the impedances of $C_{1}$ and $C_{2}$ are background signals which cause the loss of linearity and limit the resolution and the detection range of conductance detection [6,7,8,9,10,11,12,13,16,17,18,19,20,26,27,28,29,30,31,32,33,34,35].

To suppress the negative influence of the coupling capacitances, some methods have been proposed. The high-frequency method is a commonly used approach which reduces the influence of the coupling capacitances on the conductivity measurement by increasing the excitation frequency [6,7,8,9,10,11,12,13]. However, despite its usefulness, the application of the high-frequency method may cause higher requirements for the AC source and the design of the electronic circuit, and the stray capacitance is a problem which must be considered. Laugere *et al.* studied a four-electrode C^4^D sensor in which the solution measurement is realized by a differential voltage signal [17,18,30]. However, the construction is relatively complicated. Shih *et al.* and Zheng *et al.* reported a new method which is based on the parallel resonance principle [31,32]. At the resonant frequency, the negative influence of the coupling capacitances and the stray capacitance can be eliminated. Unfortunately, the resonant frequency of the circuit is related to the resistor $R_{x}$ and is difficult to determine. Kang *et al.* used the series inductance from a piezoelectric quartz crystal to compensate the conductance from $C_{1}$ and $C_{2}$ [33,34,35]. This method can effectively improve the sensitivity and the signal-to-noise ratio. However, it is mainly applied in capillaries and the operating frequency is relatively high [33,34,35]. Therefore, although many technical achievements have been obtained, more studies should be undertaken.

**Figure 2:**
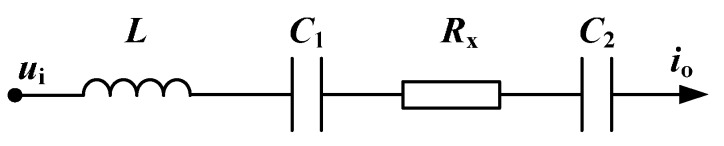
Simplified circuit of the C^4^D sensor based on series resonance.

Currently, our research group has also made some efforts in this area and a new method on the basis of the series resonance principle is proposed [26,27,28]. Figure 2 is the simplified circuit of the C^4^D sensor based on series resonance, where L is a practical inductor connected into the AC path in series. At resonance (the resonant frequency $f_{0}$ is determined by $f_{0}=\frac{1}{2\pi }\sqrt{\frac{C_{1}+C_{2}}{LC_{1}C_{2}}}$), the capacitive reactance of the coupling capacitances will be eliminated by the inductive reactance of the inductor. The reactance of the overall impedance of the detection circuit can be zero and the overall impedance only consists of the resistor $R_{x}$ which is the useful signal. Our research works have verified that with the introduction of a practical inductor, the negative influence of the coupling capacitances can be overcome, the measurement performance of the C^4^D sensor can be improved, and the C^4^D sensor can successfully implement the conductivity detection in millimeter-scale pipes [26,27,28].

However, due to the non-adjustable characteristic of a practical inductor, the C^4^D sensor based on series resonance still has its drawbacks: (1) To implement the conductivity measurement, the frequency of the AC source should be adjusted to the resonant frequency. The resonant frequency is dependent on the inductance value of the practical inductor. For a practical inductor, it is not easy to adjust its inductance value. So, the non-adjustable characteristic of the practical inductor may cause an additional requirement for the AC source, and limit (or narrow) the selection range of the AC source; (2) The non-adjustable characteristic of the practical inductor more or less limits the interchangeableness of the C^4^D sensor and, hence, causes inconvenience for the practical application of the C^4^D sensor. For example, if we hope one detection circuit can be interchangeable in different C^4^D sensors with different inner diameters when the excitation frequency is difficult to adjust or one C^4^D sensor can work at different resonant frequencies, one practical inductor is not enough and different practical inductors with different inductance values should be used to implement the resonance. Therefore, if we can seek a useful approach to overcome the non-adjustable characteristic of a practical inductor, the C^4^D sensor based on series resonance may have broader application perspective.

Fortunately, the emergence of a simulated inductor technique provides an attractive solution. As we know, according to the current technique level, it is relatively simple to reduce the dimensions of resistors, capacitances and operational amplifiers, while it is difficult to reduce the size of practical inductors [36,37,38]. Meanwhile, it is difficult to implement large-valued inductors [38,39,40,41,42]. The simulated inductor is developed and studied to satisfy the need for small-size inductors in integrated circuits. A simulated inductor is implemented by using active and/or passive components (such as resistors, capacitances and operational amplifiers) and it can function effectively to replace a practical inductor in a circuit [36,37,38]. Compared with the practical inductor, the advantages of the simulated inductor are adjustable inductance value, small size, wider range of inductance values and so on [36,37,38,39,40,41,42,43,44,45]. Currently, the simulated inductor is mainly studied and applied in integrated circuits. Our experience or knowledge on the application of the simulated inductor technique to other research fields is limited.

The aim of this work is to design an improved simulated inductor which is suitable for the C^4^D sensor based on series resonance and, hence, to develop a new C^4^D sensor. With the improved simulated inductor, the new C^4^D sensor can overcome the influence of the non-adjustable characteristic of a practical inductor on conductivity measurement.

## 2. Design of New C^4^D Sensor

### 2.1. Improved Simulated Inductor

**Figure 3:**
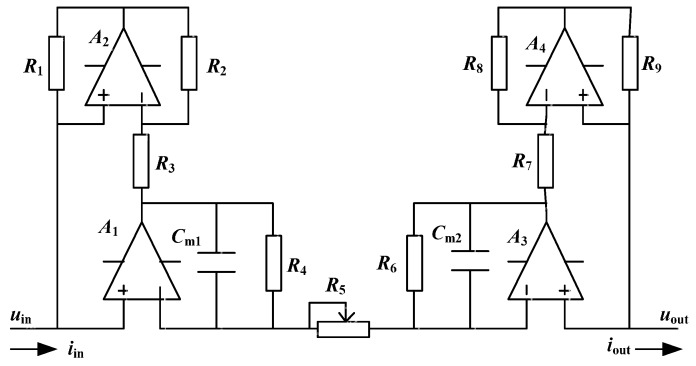
Circuit of the improved simulated inductor.

The improved simulated inductor is designed on the basis of the Riordan-type floating simulated inductor which is a classic and typical simulated inductor [36,42,44]. Figure 3 shows the circuit of the improved simulated inductor. Compared with the standard Riordan-type floating simulated inductor, the differences (or improvements) of this improved simulated inductor are as follows:

1. Considering the practical applications, to avoid the output saturation of the operational amplifiers $A_{1}$ and $A_{3}$, two resistors $R_{4}$ and $R_{6}$ are added between the inverting input and the output of the operational amplifiers $A_{1}$ and $A_{3}$, respectively.
2. The resistor $R_{5}$ is adjustable while it is an invariable resistor in the Riordan-type floating simulated inductor circuit. This modification is to make the equivalent inductance of the improved simulated inductor adjustable.

Let $R_{1}=R_{9}$, $R_{2}=R_{8}$, $R_{3}=R_{7}$, $R_{4}=R_{6}$, $C_{m1}=C_{m2}$. The impedance of the improved simulated inductor $Z_{s}$ can be described as

(1) $$Z_{s}=\frac{u_{\text{out}}-u_{\text{in}}}{i_{\text{in}}}=\frac{R_{1}R_{3}R_{5}}{R_{2}R_{4}}+j2\pi f\frac{R_{1}R_{3}R_{5}}{R_{2}}C_{m1}=r_{\text{eq}}+j2\pi fL_{\text{eq}}$$

The equivalent inductance of the improved simulated inductor $L_{\text{eq}}$ is

(2) $$L_{\text{eq}}=\frac{R_{1}R_{3}C_{m1}}{R_{2}}R_{5}$$

The internal resistance of the improved simulated inductor $r_{\text{eq}}$ is

(3) $$r_{\text{eq}}=\frac{R_{1}R_{3}R_{5}}{R_{2}R_{4}}$$

In this work, to adjust the equivalent inductance conveniently, $R_{5}$ is chosen as an adjustable resistor, while the values of the other resistors and capacitances ($R_{1}$, $R_{2}$, $R_{3}$ and $C_{m1}$) are fixed. Thus, the equivalent inductance $L_{\text{eq}}$ can be adjusted by changing the value of the adjustable resistor $R_{5}$.

### 2.2. New C^4^D Sensor

**Figure 4:**
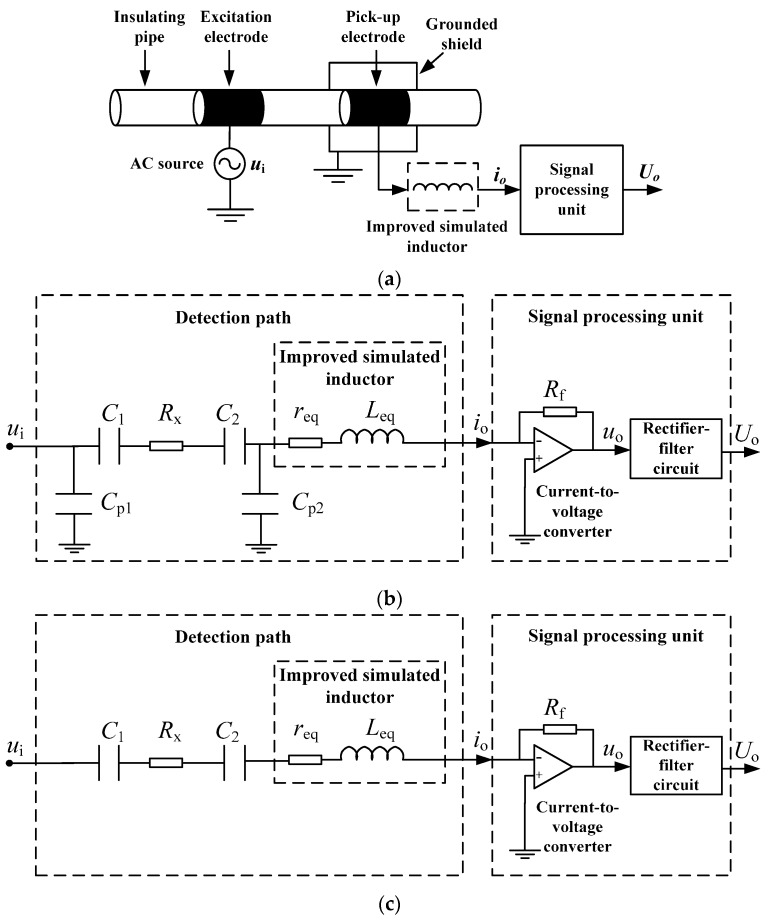
Measurement principle of the new C^4^D sensor: (**a**) Construction; (**b**) Equivalent circuit; (**c**) Simplified equivalent circuit.

Figure 4 shows the measurement principle of the new C^4^D sensor. Figure 4a is the construction of the new C^4^D sensor, including an AC source, an insulating pipe, two cylindrical metal electrodes, the improved simulated inductor, a grounded shield and a signal processing unit. The signal processing unit has two parts: a current-to-voltage converter and a rectifier-filter circuit. The improved simulated inductor is introduced to overcome the negative influence of the coupling capacitances.

Figure 4b illustrates the equivalent circuit of the new C^4^D sensor, where $C_{p1}$ is the stray capacitance arising between the excitation electrode and the grounded shield, $C_{p2}$ is the stray capacitance arising between the pick-up electrode and the grounded shield, and $R_{f}$ is the feedback resistance. Research works have verified that the stray capacitances $C_{p1}$ and $C_{p2}$ are very small and their influences can be neglected [20,26,27,28]. Thus, Figure 4b can be simplified to Figure 4c.

The overall impedance of the detection path is

(4) $$Z=R_{x}+r_{\text{eq}}+j\left(2\pi fL_{\text{eq}}+\frac{C_{1}+C_{2}}{2\pi fC_{1}C_{2}}\right)$$

where f is the excitation frequency of the AC source. According to the series resonance principle, at the resonant frequency $f_{0}$, the inductive reactance and the capacitive reactance can be eliminated by each other and the reactance of the total impedance is zero. The resonant frequency $f_{0}$ is determined by

(5) $$f_{0}=\frac{1}{2\pi }\sqrt{\frac{C_{1}+C_{2}}{L_{\text{eq}}C_{1}C_{2}}}$$

Thus, the overall impedance $Z_{R}$ at resonance is

(6) $$Z_{R}=R_{x}+r_{\text{eq}}$$

Equation (6) shows that, at the resonant frequency $f_{0}$, the influence of the coupling capacitances $C_{1}$ and $C_{2}$ can be overcome, the overall impedance only consists of the resistance elements, the resistor of the solution $R_{x}$ and the internal resistance of the improved simulated inductor $r_{\text{eq}}$. $r_{\text{eq}}$ is a background signal. The existence of $r_{\text{eq}}$ may, more or less, cause unfavorable influence on the linearity.

Furthermore, it is necessary to indicate that the reason why the improved simulated inductor is connected between the pick-up electrode and the signal processing unit (not connected between the AC source and the excitation electrode) is based on the consideration of the running stability of the simulated inductor. The components (resistors, capacitances and operational amplifiers) of the improved simulated inductor are not ideal. Additionally, the improved simulated inductor is a relatively complicated multi-closed-loop system. As we know, for a practical closed-loop system, its running stability is a problem which should be carefully considered. If the potentials of both terminals of the improved simulated inductor are unceasingly changing, it may bring some undesirable effects into the running stability of the improved simulated inductor. If the potential of one terminal could be stable, it can not only help to improve the running stability, but also help to reduce the requirement for the relevant detection circuit. As shown in Figure 4b, the output of the improved simulated inductor is connected to the inverting input of an operational amplifier (the input of the current-to-voltage converter in the signal processing unit). That means the potential of one terminal of the improved simulated inductor is stable. So, in this work, the improved simulated inductor is connected between the pick-up electrode and the signal processing unit.

## 3. Experimental Results and Discussion

### 3.1. Experimental Results

**Figure 5:**
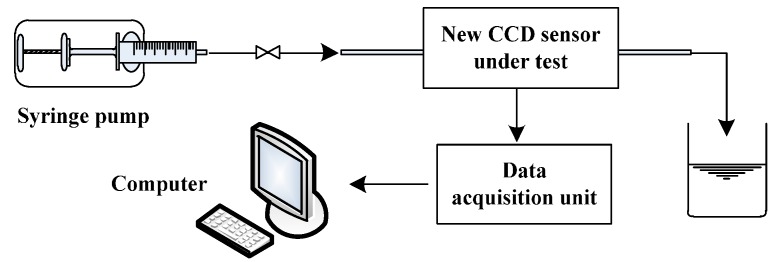
Experimental setup for conductivity measurement.

To test the performance of the new C^4^D sensor with the improved simulated inductor, the conductivity measurement experiments were carried out. Figure 5 illustrates the experimental setup for conductivity measurement. In the experiments, the insulating pipes were glass pipes. The electrodes were two rings of silver paint over the glass pipes. Three new C^4^D sensors with different inner diameters (3.0 mm, 4.6 mm and 6.4 mm, respectively) were tested. Table 1 lists the parameters of the three new C^4^D sensors. KCl solution was used as the experimental electrolyte solution and a syringe pump (Syringe Pump Model 33, HARVARD Apparatus Inc., Holliston, MA, USA, 0 mL/min~60 mL/min, ±0.35% full scale (F.S.)) was used to drive the solution into the pipe. A commercial device (cDAQ9172, National Instruments Inc., Austin, TX, USA) was used as the data acquisition unit. The reference conductivity data were obtained by a commercial contact conductivity meter (FE30, Meter Toledo Inc., Greifensee, Switzerland, 0.00 µS/cm~199.9 mS/cm, ±0.5% F.S.). The experimental temperature was around 25 °C. For the three new C^4^D sensors with different inner diameters, the improved simulated inductor was the same (its equivalent inductance value was adjusted to 53.0 mH). The components information in the improved simulated inductor circuit were: $R_{1}=R_{9}=5.1\;\text{k}\Omega$, $R_{2}=R_{8}=1\;\text{k}\Omega$, $R_{3}=R_{7}=2\;\text{k}\Omega$, $R_{4}=R_{6}=10\;\text{M}\Omega$, $C_{m1}=C_{m2}=1\;\text{nF}$, the value of the adjustable resistor $R_{5}$ ranges from 0 to 10.0 kΩ, $C_{m1}$ and $C_{m2}$ are Multilayer Ceramic Capacitor (MLCC), the amplifiers ($A_{1}$~$A_{4}$) are AD825 (Analog Devices, Inc., Norwood, MA, USA).

**Table 1:** Parameters of the three new C^4^D sensors.

New C^4^D Sensor	Length of the Electrodes (mm)	Length of the Gap (mm)	Excitation Frequency (kHz)
3.0 mm i.d. ^1^ (5.0 mm o.d. ^2^)	15.0	15.0	164.8
4.6 mm i.d. (7.0 mm o.d.)	23.0	23.0	151.7
6.4 mm i.d. (8.5 mm o.d.)	32.0	32.0	134.8

^1^ inner diameter; ^2^ outer diameter.

The relative error was adopted to assess the conductivity detection performance of the new C^4^D sensor, which is defined as

(7) $$e_{r}=\frac{\rho_{m}-\rho_{f}}{\rho_{f}}\times 100\%$$

where $\rho_{m}$ is the measurement conductivity value obtained by the new C^4^D sensor and $\rho_{f}$ is the reference conductivity value.

**Figure 6:**
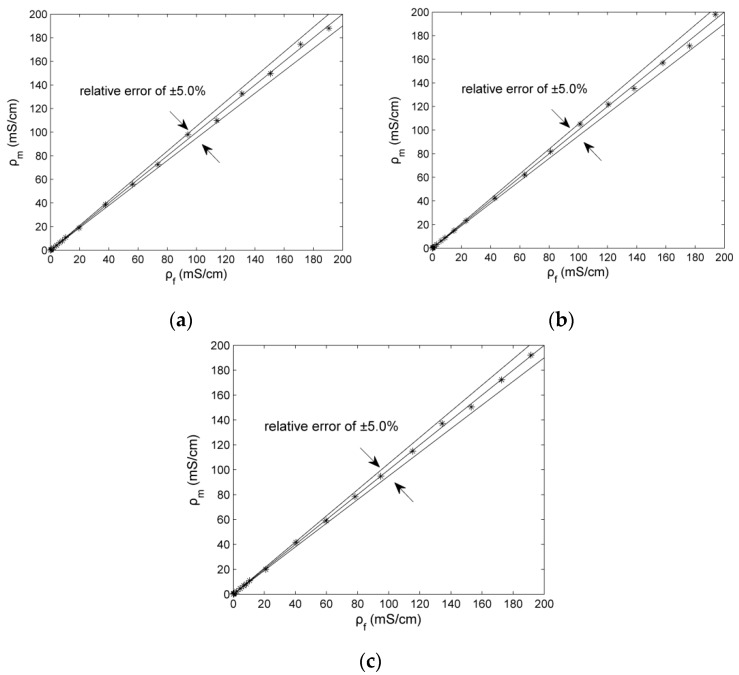
Conductivity measurement results of three new C^4^D sensors: (**a**) 3.0 mm i.d.; (**b**) 4.6 mm i.d.; (**c**) 6.4 mm i.d.

**Figure 7:**
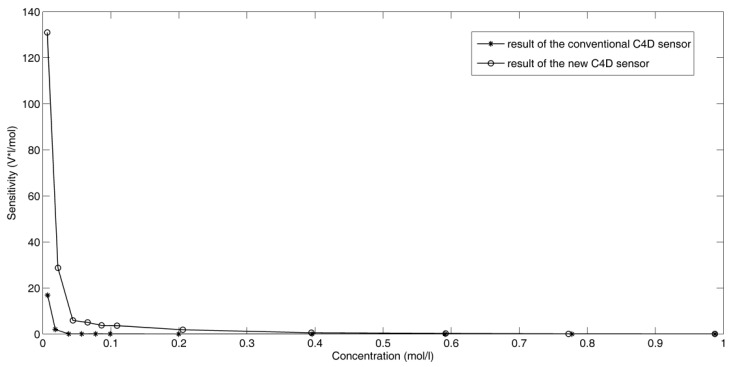
Sensitivity plots of the new C^4^D sensor with 3.0 mm i.d. and the conventional C^4^D sensor with 3.0 mm i.d.

Figure 6 shows the experimental results of the three new C^4^D sensors. Compared with the commercial contact conductivity meter, the maximum relative errors $e_{r}$ of the three new sensors are all less than 5%. Compared with the C^4^D sensors using practical inductors [26,27,28], the measurement accuracy of the new C^4^D sensor with the improved simulated inductor is comparable. Figure 7 shows the sensitivity plots of the new C^4^D sensor with 3.0 mm i.d. and the conventional C^4^D sensor with 3.0 mm i.d. Compared with the conventional C^4^D sensor, the performance of the new C^4^D sensor is better. Meanwhile, the sensitivity plots show that both sensitivities of the new C^4^D sensor and the conventional C^4^D sensor are not constant. At lower concentration, the sensitivities are relatively high while at higher concentration the sensitivities are low. Generally, the sensitivities of the new C^4^D sensor and the conventional C^4^D sensor decrease with the increase of concentration (or conductivity). The experimental results are in accordance with other researchers’ study results [12,21,22,23,24,34]. The research results indicate that the designs of the new C^4^D sensor and the improved simulated inductor are successful. The improved simulated inductor is suitable for the C^4^D sensor based on series resonance and the measurement performance of the new C^4^D sensor is satisfactory.

As mentioned in Section 2, the equivalent inductance value of the improved simulated inductor can be adjustable by changing the value of the adjustable resistor $R_{5}$. To test the adjustability of the improved simulated inductor, the relevant experiments are also performed. The test results indicate that the equivalent inductance value of the improved simulated inductor can be successfully changed from 28.8 mH to 74.1 mH by adjusting $R_{5}$ from 2.27 kΩ to 6.23 kΩ. This capability of the improved simulated inductor can greatly reduce the requirement for the AC source and is beneficial for guaranteeing the interchangeableness of the new C^4^D sensor.

**Figure 8:**
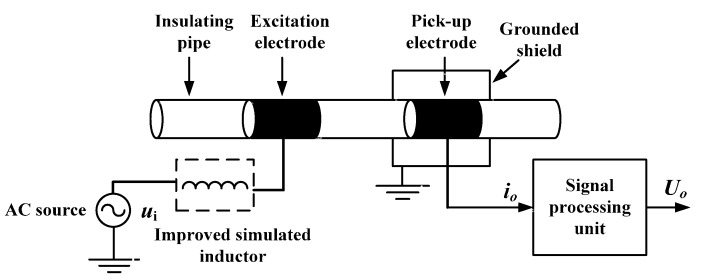
Construction of the supplementary C^4^D sensor.

**Figure 9:**
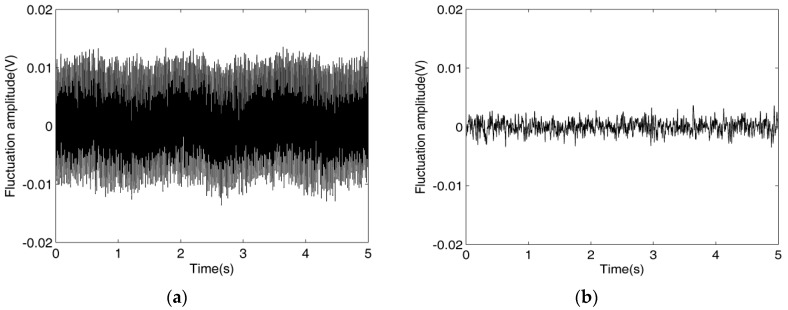
Output signals: (**a**) Supplementary C^4^D with 3.0 mm i.d.; (**b**) New C^4^D sensor with 3.0 mm i.d.

In addition, to test the reasonableness of the connection method of the improved simulated inductor, a supplementary C^4^D sensor was developed and a special comparison experiment was carried out. Figure 8 shows the construction of the supplementary C^4^D sensor. Compared with Figure 4a, obviously, unlike the new C^4^D sensor, in the supplementary C^4^D sensor the improved simulated inductor is connected between the AC source and the excitation electrode. Figure 9 shows the output signals of the new C^4^D sensor with 3.0 mm i.d. and the supplementary C^4^D sensor with 3.0 mm i.d. It is clear that the signal fluctuation amplitude of the supplementary C^4^D sensor is greater than that of the new C^4^D sensor. The special comparison experimental result indicates that, although the supplementary C^4^D sensor can implement the conductivity measurement, its running stability is obviously worse than that of the new C^4^D sensor. This experimental result verifies that our consideration of the running stability of the simulated inductor is reasonable. The connection method in the new C^4^D sensor (the improved simulated inductor is connected between the pick-up electrode and the signal processing unit) is a wise choice.

### 3.2. Discussion

The research results in this work have proved that the equivalent inductance value of a successfully designed simulated inductor can be adjusted in a relatively wide range (e.g., in this work, the equivalent inductance value of the improved simulated inductor can be changed from 28.8 mH to 74.1 mH by adjusting $R_{5}$ from 2.27 kΩ to 6.23 kΩ.). However, the adjustment range of the practical adjustable inductor is usually less than 15%. So, the simulated inductor technique indeed provides a useful approach to overcome the non-adjustable characteristic of a practical inductor. The adjustability of a simulated inductor is not only beneficial to the design of a sensor, but is also convenient for guaranteeing the interchangeableness of the sensor and its practical applications.

Meanwhile, the research results also indicate that a simulated inductor is a relatively complicated closed-loop system and its components (resistors, capacitances and operational amplifiers) are not ideal. The running stability is really a problem which should be carefully considered. According to our comparison experimental result, it is recommended that making the potential of one terminal of a simulated inductor stable is beneficial to the running stability.

Furthermore, the miniaturization of the C^4^D sensor has become an attractive research area and many researchers have made their efforts in this area [5,7,8,9,10,17,18,30]. It is necessary to indicate that, although the aim of this work is not the miniaturization of the C^4^D sensor, the research results can provide a useful reference. As we know, the practical inductor is usually a spiral inductor and its miniaturization is very difficult [36,37,38,39,40,41,42]. Unlike a practical inductor, the miniaturization of the components (resistors, capacitances and operational amplifiers) used in a simulated inductor are easy to implement [36,38,39,40]. This work actually indirectly verifies the possibility and feasibility of the miniaturization of the C^4^D sensor by using the simulated inductor technique and lays a good foundation for future research work.

## 4. Conclusions

In this work, the simulated inductor technique is introduced into the research field of C^4^D. Based on the Riordan-type floating simulated inductor, an improved simulated inductor is designed to overcome the non-adjustable characteristic of a practical inductor. With the improved simulated inductor, a new C^4^D sensor, which implements the conductivity measurement by the series resonance principle, is developed.

Three new C^4^D sensors with different inner diameters (3.0 mm, 4.6 mm and 6.4 mm, respectively) have been evaluated experimentally. The conductivity measurement results demonstrate that the design of the improved simulated inductor is effective and the new C^4^D sensor is successful. Compared with a commercial contact conductivity meter, the maximum relative error of the new C^4^D sensor is less than 5%. Compared with the C^4^D sensors using practical inductors, the measurement performance of the new C^4^D sensor is comparable.

The test experimental results show that the equivalent inductance value of the improved simulated inductor can be adjusted in a relatively wide range, compared with a practical inductor. Due to the adjustability of the improved simulated inductor, it is easier for the new C^4^D sensor to implement the conductivity measurement and the requirement for the AC source is reduced. The research results also indicate that making the potential of one terminal of a simulated inductor stable is beneficial to the running stability.

This research work verifies the effectiveness and feasibility of the application of the simulated inductor technique to the design of a new C^4^D sensor. Meanwhile, this research work expands the application fields of the simulated inductor technique and lays a good foundation for the miniaturization and integration of the C^4^D sensor in the future.
